# Continuous Publication and Other Editorial Changes

**DOI:** 10.5696/2156-9614-8.18.180610

**Published:** 2018-06-11

**Authors:** Sandra Page-Cook

**Affiliations:** Managing Editor, *Journal of Health and Pollution*, New York, NY, USA

## Abstract

**Competing Interests.** The author declares no competing financial interests.

The *Journal of Health and Pollution* (*JH&P*) announces several administrative changes and upgrades.

First, we now operate under a continuous publication workflow. Continuous publication allows us to post each article as it is ready, rather then waiting for the ‘right time’ to publish each quarterly edition. We will still group articles together into a quarterly edition in March, June, September and December, but an article that is ready in April will not need to wait for the June edition to be published. In this new model, an article would be published under the June ‘Issue in Progress’. Continuous publication will further decrease the time from submission to publication. Articles posted in advance will appear in the ‘Issue in Progress’ link, and are final, reviewed and edited articles. Individual articles will be promoted on social media as they are published, thus giving each article greater attention. We also encourage authors to promote their own work on social media.

Second, and associated with moving to a continuous publication workflow, each article will begin with page 1, and will be identified by a unique Citation Identifier (CID). The CID is a six-digit number that will contain the year, month and article number. For example, in this edition, the June 2018 edition, all CIDs begin with 1806. The articles will not necessarily appear in numerical order by CID in the final edition, nor will the CID define the article type (Research, Review, Case Study, etc.)

Third, and finally, we've also taken this opportunity to introduce a fresh look to our cover. We no longer need to include a Table of Contents on the PDF cover, so enjoyed some creative freedom to make a changewhat do you think?

We believe these are all positive changes. Please send any feedback on these new processes to sandy@pureearth.org. Thank you for supporting the Journal of Health and Pollution.

